# Supplementary material for: Are carbon emissions trading and green financial instruments synergistic? -Comprehensive quantitative research based on content analysis
Source: PLoS One. 2024 Mar 7;19(3):e0298601. doi: 10.1371/journal.pone.0298601 (PMC10919601; doi:10.1371/journal.pone.0298601)
Supplement: S1 Data — (ZIP) [file pone.0298601.s001.zip › Data/地级市Z_F工作报告.zip!/║╙─╧/σo╤⌠╩╨/└┤╫╘╣1⁄2╓┌║┼í╛┬φ┐╦╩2╛▌═°í┐_2009.docx]

各位代表：

现在，我代表市人民政府，向大会作政府工作报告，请予审议，并请政协各位委员提出意见。

一、过去五年工作回顾

2004年以来，本届政府在省委、省政府和市委的正确领导下，坚持以科学发展观统领经济社会发展全局，团结带领全市人民继续解放思想，深化改革开放，推动科学发展，促进社会和谐，圆满完成了市五届人大确定的各项目标任务，全市主要经济社会发展指标实现翻番，各方面工作都取得新的进展。市政府连续四年被省政府授予目标管理优秀单位称号。

——总体经济实力迈上新台阶。初步核算，2008年，全市生产总值657亿元，是2003年的2.52倍。人均生产总值18803元，是2003年的2.54倍。规模以上工业实现利润112亿元，是2003年的8.6倍。财政一般预算收入25.3亿元，是2003年的2.47倍。

——农业、工业和服务业持续发展。粮食总产244.8万吨，比2003年增加84.5万吨。全部工业增加值400.2亿元，第三产业增加值129.3亿元，分别是2003年的3.1倍和2.2倍。二、三产业增加值占生产总值的比重达到86.2%，比2003年提高2.6个百分点。

——投资、消费和出口同步增长。全社会固定资产投资334.6亿元，社会消费品零售总额166.2亿元，出口总额3.4亿美元，分别是2003年的3.3倍、2.2倍和4.9倍。

——人民生活进一步改善。城镇居民人均可支配收入12731元，农民人均纯收入4065元，分别是2003年的2倍和2.1倍。 ——社会事业全面发展。基础教育进一步巩固，职业教育、继续教育和高等教育有了新的发展。科技进步贡献率明显提升。公共卫生体系不断健全。文化体育事业繁荣发展。社会保障体系进一步完善。人口自然增长率连续五年低于全省平均水平。

五年来，我们突出抓了以下重点工作：

（一）加快产业结构调整和发展方式转变。深入实施以工兴市战略，按照“龙头带动、产业支撑、优化结构、集群发展”的思路和新型工业化要求，大力培育石油化工、林纸林板、玻璃及电光源、食品加工、冶金建材、机械设备制造、纺织和电力等八大优势产业，将石油化工产业作为战略支撑产业来抓，支持中原油田、中原乙烯、中原大化、龙宇化工等一批石油化工重点企业拉长产业链条，提升产业竞争力。围绕石油化工、煤化工、盐化工“三化”链接，谋划实施了中原大化煤化工、龙宇煤化工、永龙盐化工等一批大项目。着力打造濮阳经济开发区、濮阳工业园区等8个产业集聚区和河南省电光源产业基地。五年累计新上投资超千万元的工业重点项目525个，总投资345.5亿元。2008年，全市规模以上工业增加值完成346亿元，是2003年的3.4倍；八大优势产业完成工业增加值312.2亿元，占全市规模以上工业增加值的90%；全市“双超”企业254家，比2003年增加229家。立足优化产业结构，加快建立现代产业体系，文化、旅游、现代物流等服务业全面发展。积极推进文化体制改革，文化产业发展迈出新步伐。市杂技艺术中心列入全省100户高成长服务企业名单。华晨杂技集团被授予“国家文化出口重点企业”。旅游业整体水平明显提高，我市被评为“中国最佳文化生态旅游城市”。2008年，旅游收入3.4亿元，是2003年的2倍。房地产业发展迅速，建筑业总产值、房地产开发投资年均分别增长21.5%、35.2%。金融业平稳健康运行，服务经济发展能力进一步增强。大力实施科教兴濮战略，着力推进自主创新，加快以企业为主体的技术创新体系建设，加强市校、市院合作，实施了一批重大科技专项，科技创新支撑和引领濮阳产业结构调整的能力持续增强。五年共实施国家级科技计划项目37项、省级科技计划项目149项。建立国家级企业技术中心1家、省级工程技术中心3家、省级企业技术中心15家。着力推进节能减排和生态建设。严格实行节能减排问责制，加快淘汰落后生产能力。关停小火电机组8台。关闭取缔“十五小土”企业105家。实施了金堤河、濮水河、马颊河综合治理工程，对金堤河水质严重污染问题进行了重点治理。拆除实心黏土砖瓦窑厂326座，复垦土地1118公顷。实施了龙丰热电脱硫等一批环保基础设施工程，各县污水处理厂和垃圾处理场实现达标运营。开工建设了国电中水回用工程。2008年全市单位生产总值能耗下降4.6%，化学需氧量、二氧化硫排放量分别削减5.1%、8.6%。创建国家生态示范区工作通过省考核验收。

（二）推进社会主义新农村建设。把解决“三农”问题作为全部工作的重中之重，以推进农业产业结构调整为抓手，以发展现代农业为重点，千方百计促进农业不断增效、农村加快发展、农民持续增收。认真落实各项支农惠农政策。2004年以来全市共发放各类惠农补贴8.5亿元。加强基本农田保护，连续十年实现耕地占补平衡。农业结构调整步伐大大加快。全市优质小麦、玉米、水稻播种面积分别达到232万亩、100万亩、64万亩，建立国家级农业示范区7个、无公害农产品基地47个。全面推进林业生态市建设，速生丰产林面积达到130余万亩，全市林木覆盖率27.3%，比2003年提高5.3个百分点。发展林下经济10万亩。成功举办了第23届国际杨树大会濮阳现场会。全市养殖小区145个，各类规模养殖场1.56万个。积极推进农业产业化经营。农业产业化龙头企业420家，比2003年增加167家，其中年产值超亿元的14家，增加6家。农业农村基础设施建设不断加强。荣获全省“红旗渠精神杯”市级杯九连冠。解决了41万农民的饮水安全问题。建设农村户用沼气池8.7万座。建设便民超市1888个。大力开展“学习李连成，建设社会主义新农村”活动，按照“示范带动、组织推动、产业强村、项目富民”的思路，积极推进新农村建设，涌现出市级典型村306个、县级典型村500个，形成了3条新农村建设示范带。

（三）夯实基础设施和基础产业。五年累计新上重点项目730个，建成投用616个，完成投资547.6亿元。基础设施方面，濮鹤高速、大广高速濮阳段、安南高速建成通车，汤台铁路升级改造工程完工，濮范高速掀起建设高潮，大广高速豫冀省界至南乐段开工建设。濮阳新一代天气雷达站、5个县城污水处理厂和垃圾处理场等项目建成投用。新建黄河防洪工程项目12个。黄河标准化堤防工程、金堤河张庄提排站改扩建工程、市城市生活垃圾填埋场等项目加快建设，市城区至工业园区快速通道可研报告通过省发改委评审。基础产业方面，国电濮阳热电2×20万千瓦机组建成并网发电，中原大化50万吨甲醇、龙丰纸业10.8万吨杨木化机浆及25万吨轻量涂布纸、龙宇化工20万吨甲醇改扩建、同力水泥100万吨水泥粉磨站等项目建成投产。永龙化工20万吨烧碱25万吨聚氯乙烯、北京林氏集团5万吨聚异戊二烯乳液等项目开工建设，龙丰纸业15万吨浆40万吨轻量涂布纸、国电濮阳热电2×60万千瓦机组、龙丰热电60万千瓦机组、中原乙烯20万吨烯烃等项目正在开展前期工作。

（四）统筹城乡协调发展。一是围绕巩固和发展城市创建成果，以规划为龙头，加强城市建设和管理，中心城市的辐射带动力明显增强。完成了第三轮城市总体规划修编，编制了历史文化名城保护等专项规划和中心商务区等控制性详细规划。投资18亿元，建成了黄河路东延、京开大道北入市口拓宽改造等20多个重点城建项目；新修、拓宽城市道路40条80多公里；城区西北环路改建工程建成通车，结束了我市环城公路不通的历史。吸引社会资金启动了“城中村”改造。二是全面推进新一轮村镇规划修编工作，完成了5个县城的总体规划和重点镇规划。清丰县城、范县新区等5个县城建设取得明显成效。全市城镇化率33.8%，比2003年提高8.2个百分点。三是完善激励机制和考评体系，引导县（区）立足优势，大力发展县域经济。2008年全市县域生产总值完成459亿元，县均财政一般预算收入1.4亿元，分别是2003年的3.2倍、2倍。清丰县、濮阳县先后被评为全省县域经济发展先进县。

（五）深化改革开放。重点领域和关键环节改革实现突破。全市地方国有及国有控股企业268户，改制面98%，盘活国有资产18.8亿元，引进社会资金19亿元，安置职工2.8万人。引进中国国电集团、河南煤业化工集团、省投资集团、香港嘉联集团、立马集团、上海爱普公司等战略投资者，对市甲醇厂、市氯碱厂、市三强电力集团、市啤酒厂、市橡胶粉厂等企业成功实施了兼并重组和收购。完成了乡镇机构改革、国有粮食购销企业改革、农村信用社体制改革，农村公路管理养护体制、统计方法制度、城市公用事业、水利管理养护体制、财税金融、价格、投资等各项改革均取得了阶段性成果。对外开放水平不断提高。通过赴“珠三角”、“长三角”、“环渤海经济圈”等地自主招商和参加省组织的招商活动，引进市外资金项目904个，总投资551.7亿元，实际利用市外资金191.2亿元，其中引进1亿元以上的项目47个。止2008年底，已建成投产699个，完成投资328亿元。成功举办了世界张氏总会第二届恳亲大会、世界舜裔宗亲联谊会第十九届国际大会、濮阳第五届中华龙文化节暨第二届杂技艺术节等活动，签约项目70个，总投资82.9亿元。我市与吉尔吉斯共和国楚河区正式确立国际友好城市关系。外经三项指标总额连续十年保持全省第一。非公有制经济快速发展。止2008年底，全市规模以上非公有制企业873家，其中年营业收入超千万元的728家，超亿元的194家。全市非公有制经济完成增加值349亿元，占全市经济总量的53.1%，比2003年提高17.7个百分点。

（六）着力解决关系人民群众切身利益的问题。大力组织财政收入，努力争取资金，优化财政支出结构，财政保障能力明显增强，有力支持了农业、教育、科技、社会保障等法定和重点支出，促进了各项事业进步。集中力量解决重大民生问题。筹集资金，五年完成了向人民群众承诺的58件实事，解决了一批涉及民生的突出问题，特别是两个关系濮阳发展全局和民生的重大问题。一是完成了渠村引黄闸改建工程，解决了200万城乡居民用水、城市工业用水安全和193万亩耕地灌溉问题。二是建设了金堤河除涝工程，先后建成了10座支沟口排涝闸站，基本消除了范县、台前县60万亩农田的内涝危害。就业再就业工作成效显著。全市城镇新增就业人员31.9万人，下岗失业人员再就业12.3万人，其中“4050”人员5.5万人，城镇登记失业率均控制在4%以内。发放小额担保贷款3.6亿元，实现成功创业1.2万人，带动2.9万人实现就业再就业。社会保障体系不断完善。企业职工基本养老保险按时足额发放。城市低保和农村低保实现应保尽保。对75所敬老院进行了改扩建，全市五保对象集中供养率42%。建设经济适用房103万平方米，为城市低收入家庭提供了9414套经济适用房。发放廉租住房补贴资金405万元，对城市住房困难家庭实施保障3931户。发放住房公积金贷款3.8亿元，支持城镇低收入家庭购建住房120万平方米。扶贫开发和救灾工作成效显著。投入1.4亿元，完成214个贫困村整村推进建设任务，20.1万贫困人口解决和巩固了温饱。新改建农村公路2470公里，通车里程是2003年的2.2倍。全市2966个行政村全部实现村村通油路或水泥路、村村通班车。积极开展支援四川灾区抗震救灾工作，圆满完成了国家、省下达的各项任务。各项社会事业全面发展。认真落实省“兴豫之光”行动计划，引进高层次人才工作取得显著成效。选派了3名博士担任县（区）长助理，17名硕士担任乡（镇）长助理，选聘了901名大学生任村干部。建设了市、县级疾病控制中心，市、县传染病医院、传染病区，市120紧急救援中心和市中心血站。改造建设了75座乡镇卫生院。社区卫生服务覆盖率91%。实施了新型农村合作医疗制度，全市参合农民覆盖率88.3%。全市城镇居民医疗保险参保登记人数25.95万人，覆盖率70%。投资1.1亿元，新建、改扩建了23所城区中小学。改造农村中小学D级危房59.7万平方米，新建校舍56.3万平方米。投入“两免一补”资金5.7亿元，惠及农村中小学生385.7万人次。免除了城市义务教育阶段学生学杂费。濮阳职业技术学院实现了“千亩校园，万人大学”阶段性目标，办学水平明显提高。市文化艺术中心、综合活动中心等标志性文化设施建成。新建了一批乡镇综合文化站。我市被命名为“国家历史文化名城”。濮阳县被评为“河南省十大文化强县”。成功举办河南省首届杂技“百戏奖”暨第七届杂技大赛和“中国·范县郑板桥文化节”。大弦戏、东北庄杂技列入国家级非物质文化遗产保护名录。围绕稳定低生育水平，扎实开展优质服务活动，清丰县、南乐县被评为“国家计划生育优质服务先进县”，濮阳县、范县被评为“省计划生育优质服务先进县”。累计向全市计生户发放奖扶金1107万元。全民健身运动和竞技体育运动积极开展。扎实推进城市爱国卫生管理，通过了国家卫生城市复检。积极开展“家电下乡”工作，销售“家电下乡”产品4.6万台（部），向农户发放补贴607万元。开展了第二次全国农业普查、第二次全国经济普查等大型调查工作。市档案馆晋升国家一级综合档案馆。首部《濮阳市志》出版。《濮阳年鉴》被评为首届中国地方志年鉴奖特等奖。安全生产和社会稳定得到加强。加强食品药品安全监管，妥善应对问题奶粉事件，实现了无群体事件、无医疗事故、无死亡病例的目标。安全生产工作成效显著，连续五年被评为“全省安全生产工作优秀市”。强化社会治安综合治理，严厉打击各类违法犯罪活动，加强信访、民族宗教工作，建立健全应急机制，社会大局保持稳定，连续五年被评为“全省平安建设先进市”、“全省信访工作先进市”。重视国防后备力量建设，国防教育、国防动员、人民防空、民兵预备役建设得到加强，军政军民团结。广播电视、发展研究、机关事务管理、残疾人事业、审计、地震、气象、保险、烟草、邮电通信等工作都取得了新成绩。

（七）加强精神文明和民主法制建设。围绕建设社会主义核心价值体系，加强社会主义荣辱观教育，涌现出李全芳、崔运宗等一大批先进典型。继续推进精神文明创建活动，再次被命名为“全国创建文明城市工作先进城市”。深入开展“道德规范进万家、诚实守信万人行”活动。未成年人思想道德建设及大学生思想政治教育工作进一步加强。广泛开展革命传统和理想信念教育，清丰单拐革命旧址被确定为“全国爱国主义教育示范基地”。坚持依法行政，自觉接受人大法律监督、工作监督和政协民主监督，认真办理人大代表议案、建议和政协提案，支持各民主党派、工商联民主监督、参政议政。认真贯彻《全面推进依法行政实施纲要》，全面推行行政执法责任制，依法规范行政权力运行。加强农村基层民主法制建设，顺利完成第五届、第六届村委会换届选举。深入实施“治庸计划”，行政效率和服务水平进一步提高。认真落实党风廉政建设责任制，反腐倡廉取得新成效。

过去五年，我们在实践中积累了宝贵经验。主要是我们坚持解放思想、实事求是，从濮阳实际出发，大力实施以工兴市战略、科教兴濮战略、人才强市战略、开放带动战略、可持续发展战略和品牌城市战略，大大增强了濮阳发展的活力和动力；坚持以科学发展观统领政府工作全局，把结构调整和转变发展方式作为经济工作的主线，统筹城乡协调发展，大力发展工业、农业和第三产业，积极培育八大优势产业，不断提高经济发展的质量和效益；坚持执政为民，把改善和保障民生、为人民谋利益作为政府工作的出发点和落脚点，做到发展为了人民，发展依靠人民，发展成果由人民共享；坚持求真务实，深入推进“治庸计划”，加强行政效能建设，转变工作作风，优化发展环境，确保中央和省、市的各项决策部署落到实处。

各位代表，过去五年所取得的成绩，是省委、省政府和市委正确领导的结果，是全市人民共同奋斗的结果。在此，我代表市人民政府向全市工人、农民、知识分子、干部、人民解放军指战员、武警官兵、公安民警以及各界人士致以崇高的敬意，表示衷心的感谢！

同时，我们也清醒地看到，濮阳经济社会发展中还存在不少矛盾和问题。主要是受国际金融危机严重影响，市场有效需求不足，企业经营困难加剧，经济下行和财政保障压力加大。加上长期积累的城乡二元结构矛盾、农业基础比较薄弱、农业现代化程度不高、县域经济实力不强、产业集聚区发展滞后、缺乏战略支撑项目等问题，实现经济平稳较快发展面临更大的困难。此外，经济发展与节能减排的矛盾日益突出；农民持续增收难度加大；就业形势严峻；社会保障压力增加；个别单位和领导干部、办事人员思想不够解放，大局意识、服务意识不强，工作效率低下；经济发展环境还需进一步优化等等。对于上述问题，我们一定高度重视并认真解决。



二、政府工作的总体要求、奋斗目标和工作重点

做好今后五年特别是2009年政府工作，必须正确认识和把握当前经济形势。首先，我们必须深刻认识国内外经济形势的严峻性和复杂性。今年可能是新世纪以来经济发展最为困难的一年。当前国际金融危机尚未见底，对实体经济的影响不断加深，全球经济增长明显减速，经济增速下滑压力加大；世界金融危机外部影响和我国经济周期性调整叠加重合，短期问题与长期矛盾相互交织，需求不足和竞争加剧双重挤压，一些深层次矛盾更加凸显，使得我市面临的形势非常复杂、十分严峻。其次，我们还要看到面临的重大发展机遇。我市与全国、全省一样仍处在重要战略机遇期，经济发展的基本态势没有因为国际金融危机影响而逆转；全球经济深刻调整必然促进国内外产业转移重组，有利于我们加快培育主导产业，有利于我们加快产业集聚区建设，有利于我们加快产业结构调整，有利于我们培育新的经济增长点；国家实施积极的财政政策和适度宽松的货币政策，加大对中西部地区和“三农”投入力度，有利于我们借力加强基础设施建设和民生项目建设，有利于我们加强薄弱环节，有利于我们解决长期以来没有能力解决的问题。第三，我们更要看到濮阳发展的巨大潜力。建市以来，历届市级领导班子团结带领全市人民，艰苦创业，开拓进取，探索出一条符合濮阳市情的发展道路，创造了辉煌的业绩。本届政府突出实施以工兴市战略，统筹城乡协调发展，扎实推进新农村建设，不断完善基础设施，全面推进各项社会事业，濮阳经济社会健康快速发展，经济总量、发展速度、增长质量都上了一个新台阶，已具备了加快发展的基础和必要条件。特别是目前全市上下人心思进，发展愿望强烈，已经形成了干事创业的浓厚氛围，这是我们克难攻坚，乘势而上的强大动力。展望未来，我们对濮阳的发展充满希望和信心。因此，我们既要增强忧患意识，更要增强机遇意识，以对濮阳人民高度负责的态度和勇于战胜困难的精神，全力促进濮阳经济平稳较快发展。

各位代表，今后五年是全面建设小康社会、建设富裕文明和谐新濮阳的关键时期。我们必须积极适应国内外形势变化，全力抓住并用好战略机遇期，以科学发展观统领经济社会发展全局，力争经过五年的发展，使濮阳综合经济实力显著提高、发展方式进一步转变、区域竞争力和可持续发展能力明显提升。

——经济强市建设实现新跨越。2013年，力争全市生产总值达到1000—1200亿元，人均生产总值达到4000—5000美元。坚持以工兴市不动摇，大力培育八大优势产业和战略支撑产业，加快工业化进程；强力推进农业现代化，新农村建设取得重大突破；千方百计推动服务业发展，促其成为新时期重要的经济增长点。 ——城乡统筹建设取得重大进展。科学编制城乡规划，完善城市基础设施，严格城市管理，提升城市品位，中心城市的区域带动能力和核心竞争力显著增强。大力发展县域经济，进一步加强县城和小城镇基础设施建设，促使人口和资源合理聚集，加快城镇化建设步伐，经过五年的努力，力争全市城镇化率达到全省平均水平。

——文化强市建设迈出重要步伐。文化资源优势得到充分发挥，文化事业和文化产业加快发展。抓住机遇，加快完善公共文化服务体系，全面改善基层公共文化服务设施，大力提高公共文化产品服务和供给能力；重点打造杂技、乒乓球、休闲旅游等特色文化产业，文化软实力不断增强，全社会文明素质明显提高。 ——和谐濮阳建设扎实推进。不断增加城乡居民收入，进一步提高生活水平和质量。坚持改革发展成果人民共享，基本公共服务趋于均等化。实现城乡义务教育均衡发展，提升高等教育办学层次和质量。继续稳定低生育水平，改善出生人口结构，提高出生人口质量。着力改善基本医疗卫生服务，扩大劳动就业，初步建立覆盖城乡居民的社会保障体系，基本消除绝对贫困现象。 ——生态文明建设取得阶段性成效。着力提升能源资源利用效率，大力发展循环经济，主要污染物排放量得到有效控制，生态文明观念深入人心。到2013年单位生产总值能耗比2008年降低18%左右。林业生态市初步建成，林地面积达到200万亩，林木覆盖率达到30%以上。生态环境质量明显改善。经济发展与人口、资源、环境趋于协调。

各位代表，今年是新一届政府的开局之年，各项工作都要开好头，起好步。今年政府工作的总体要求是：以邓小平理论和“三个代表”重要思想为指导，深入贯彻落实科学发展观，突出实施以工兴市战略，加快社会主义新农村建设步伐，把保持经济平稳较快发展作为经济工作的首要任务，坚持扩内需、保增长，调结构、上水平，抓改革、增活力，重民生、促和谐，促进经济建设、政治建设、文化建设、社会建设、生态文明建设协调发展。今年经济社会发展的主要目标是：生产总值增长10%；地方财政一般预算收入增长9%；全社会固定资产投资增长20%；社会消费品零售总额增长14%；外贸出口增长10%；实际利用外资7500万美元；居民消费价格涨幅控制在4%左右；城镇居民人均可支配收入实际增长8%；农民人均纯收入实际增长8%；单位生产总值能耗降低4.56%，化学需氧量、二氧化硫排放量分别控制在2.56万吨、2.93万吨以内；人口自然增长率控制在6.5‰以内；城镇新增就业5万人，城镇登记失业率控制在4.5%以内。完成今年的目标任务，要重点抓好八个方面的工作：

（一）集中力量扩大市场需求，保持经济平稳较快发展。一是千方百计抓好重大项目建设。抢抓国家、省扩内需、保增长的政策机遇，围绕国家、省重点支持的基础产业和基础设施建设领域，积极主动地申报项目，争取资金，谋划建设一批对濮阳发展起战略支撑作用的重大项目，充分发挥投资对经济增长的拉动作用。今年要重点抓好基础产业和基础设施方面投资超千万元的项目140个，其中投资超亿元的项目30个、超5亿元的项目8个、超10亿元的项目3个，年内计划完成投资90亿元。产业升级方面：加快永龙化工20万吨烧碱25万吨聚氯乙烯、永乐化工10万吨L—乳酸、北京林氏集团5万吨聚异戊二烯乳液、通宇纸业5万吨轻量涂布纸、武汉力诺40万台太阳能热水器二期、濮耐公司连铸三大件等项目建设；开工建设恒润石化聚异戊二烯装置等项目；积极推进龙丰纸业15万吨浆40万吨轻量涂布纸、龙宇化工120万吨甲醇改扩建、中原大化30万吨合成氨原料线路改造、中原乙烯20万吨烯烃和范县、清丰县、濮阳县盐化工等项目前期工作。能源方面：建成榆林—濮阳—济南天然气管道工程濮阳段、濮阳西郊220千伏输变电站等项目；加快电网运行和生产指挥基地等项目建设；做好国电濮阳热电2×60万千瓦机组、龙丰热电60万千瓦机组等项目前期工作，争取尽快开工建设。交通方面：加快濮范高速、大广高速豫冀省界至南乐段等项目建设；配合做好山西中南部铁路通道濮阳段建设；积极推进德商高速范县段等项目前期工作。农林水方面：加快金堤河张庄提排站改扩建、黄河标准化堤防二期工程等项目建设；积极实施渠村灌区、南小堤灌区续建配套与节水改造工程；加快建设大型商品粮基地等一批项目。二是着力扩大消费需求。努力增加城乡居民收入。推进事业单位工资制度改革，落实企业最低工资制度，健全农民工工资支付保障机制。落实好困难群众社会救助政策。加大对家庭经济困难学生补助力度。积极培育新的消费热点。引导房地产企业开发建设中低价位、中小套型的普通商品房，鼓励大型房地产企业进行集中连片开发，促进房地产市场稳定健康发展。加快发展住宿餐饮、社区商业、物业、家政等服务性消费，开发与节假日调整相适应的旅游、文化、体育健身和网络等热点消费。进一步优化消费环境。落实居民购买汽车、住房等大宗消费的鼓励政策，全面清理消费领域相关规费。落实“家电下乡”、“农机下乡”和“汽车、摩托车下乡”补贴政策。坚决打击制售假冒伪劣商品和价格欺诈等违法行为，维护消费者合法权益。大力发展现代服务业。落实扶持政策，支持文化、旅游等现代服务业发展。搞好城区商业网点规划，增加城区市场网点。建立健全流通信息网络，推动现代物流业加快发展。积极拓展新型服务领域，发展面向民生的服务业。三是努力保持对外贸易稳定增长。支持纺织、农产品等劳动密集型产品出口，扩大装备制造、耐火材料、机电、化工等高新技术产品出口，提高高附加值产品出口的比例。加快做好商检办事机构规划建设工作，争取在我市设立海关办事机构。四是全面落实财政金融政策。落实积极的财政政策和适度宽松的货币政策，综合运用多种手段，扩大政府投资，引导带动社会投资；依法加强税收征管，规范非税收入管理。优化支出结构，确保工资发放、法定支出、民生项目配套等资金需要。落实金融促进经济发展的政策措施，增加信贷投放，支持企业发展。按照省政府要求，建立以财政为依托的县级中小企业担保机构。进一步做好城市信用社和农村信用社改革工作。加强和改进金融监管，营造良好的金融生态环境。

（二）突出抓好工业结构调整和产业优化升级，强力推动工业经济快速发展。一是培育壮大优势产业和战略支撑产业。深入实施以工兴市战略，支持八大优势产业引进战略投资者，新上一批科技含量高、有自主知识产权并带动产业发展的龙头项目。着力抓好中原油田油气勘探工程、永龙盐化工等84个重点工业项目建设，壮大工业经济实力。围绕“三化”链接，规划建设一批重大化工项目，加快培育工业经济发展的战略支撑点。积极主动地帮助中原油田、中原乙烯、中原大化、龙宇化工等重点企业应对危机，战胜困难，发展壮大。年内全市全部工业增加值达到448亿元以上，增长12%以上；规模以上工业增加值达到391亿元以上，增长13%以上。二是加快产业集聚区建设。从统筹发展城市经济和县域经济出发，立足区域资源优势和地方产业特色，明确功能定位，创新体制机制，营造良好环境，强力推进产业集聚区建设，培育壮大特色主导产业，形成新的“濮阳名片”，产生产业集聚的“滚雪球”效应。完善道路、供排水、供电、供气、污水垃圾处理、通信网络等基础设施建设，配套社会服务体系和公共服务管理，为项目入驻和人口集聚创造条件。按照突出特色、培育集群、形成链条、实现循环的原则，引导八大优势产业、高新技术产业和重点项目向产业集聚区布局，谋划实施一批关联度高、辐射力大、带动力强的龙头型、基地型项目。三是开展“企业服务年”活动为企业排忧解难。按照《濮阳市企业服务年活动实施方案》要求，全面落实各项优惠政策，积极推行现场办公、特事特办、限时办结等服务措施，完善项目协调推进机制，在重大项目建设、拓宽融资渠道、清理涉企收费等方面加大帮扶力度，重点帮扶100家骨干企业。支持企业开展各种促销活动，大力开拓市场。继续组织企业、项目单位开展银企洽谈、供需对接等多种活动，实现银企双赢。同时，加强经济运行调控调节，搞好煤电油气运和土地、资金调节，努力保障经济发展要素资源的基本供给。四是积极推进企业自主创新能力建设。全面贯彻落实全省加快科技创新促进产业发展的意见，加快构建自主创新体系，建设科技企业孵化器，重点支持国家级、省级技术研发中心建设，加快构建以企业为主体、市场为导向、产学研相结合的技术创新体系，培育壮大高新技术产业。大力实施人才强市战略，充分利用国际金融危机期间人才流动加快的形势，加强各类人才的引进工作，为经济发展提供人才和智力支撑。五是加强节能减排和生态建设。大力促进节能降耗，突出抓好工业、交通、建筑等关键领域节能工作。加快环保基础设施建设，加快市、县环境自动监控系统建设，争取上半年完成全市城市污水处理厂化学需氧量和氨氮自动监控设备更新改造，确保市、县污水处理厂达标运营。加强对濮水河等河流环境综合整治，对造纸、制药、羽绒等行业企业实施深度治理，巩固国家生态示范区创建成果。大力发展循环经济，培育循环经济产业链，建设循环经济试点园区和试点企业。加强对企业的节能减排指标体系、监测体系和考核体系建设，实现节约、清洁、可持续发展。

（三）巩固和加强农业基础地位，扎实推进新农村建设。一是加大对“三农”的投入。落实好国家和省各项强农惠农政策措施，各级财政对农业投入增幅高于经常性收入增幅，较大幅度提高粮食最低收购价，提高农资综合直补标准，扩大良种补贴和农机具购置补贴。二是进一步提高粮食综合生产能力。按照省统一部署，组织实施粮食核心区规划建设，实现粮食持续增产。抓住省建设濮阳大型商品粮基地的机遇，改善生产条件，稳定播种面积，优化品种结构，开展高产创建活动，确保我市粮食生产稳定在200万吨以上。三是加快发展现代农业。大力发展以优质小麦、优质水稻、优质玉米为主的优质粮食生产，扩大食用菌、花卉、尖椒、小杂果等特色经济作物种植面积，大力发展林下经济。抓住省重点支持肉牛、肉羊、生猪主产县和黄河滩区绿色奶业示范带发展的机遇，积极推广生态环保养殖模式，建设一批标准化规模养殖场和养殖小区，加快优质畜产品生产及加工基地建设。重点扶持家家宜米业等13家省级和60家市级重点农业产业化龙头企业做大做强。加强新型农业科技推广和科教服务体系建设，完善农畜产品质检体系和动物疫病防控体系。加强气象灾害信息预警预报和人工影响天气业务系统建设，增强防御自然灾害的能力。加快林业生态市建设，实施一批国家、省、市林业重点生态工程。四是完善农业农村基础设施。因地制宜，分类指导，深入开展“学习李连成，建设社会主义新农村”活动。积极争取国家和省的支持，调整财政支出结构，抓好农田水利和农村水、电、路、气等基础设施建设，筹措资金9000万元，新改建农村公路220公里，其中县乡道路100公里，村道120公里。推进农业水利化、农田配套化、农业机械化进程。实施村镇绿化工程，创建一批省级生态文明村。加快发展农村社会事业，进一步改善农村生产生活条件。五是深化农村改革。按照中央、省统一部署和要求，积极稳妥地推进农村各项改革，促进农业农村发展和农民增收。

（四）坚定不移地推进改革开放，为科学发展提供体制机制保障和强大动力。一是加快国有企业改革和战略重组。继续深化国有企业改革，建立和完善现代企业制度。对一般竞争性企业和各类中小企业实行分类指导，加快改制退出步伐，年内完成市直局委所属企业脱钩改制。继续做好市林钢公司、三胺化工等工业企业的改革工作。加快推进国有非工业企业改革步伐。集中力量抓好与河南煤业化工集团、省投资集团等战略合作者已签订合作项目的实施。千方百计争取与更多的世界500强企业和国内知名企业签订战略合作协议，对我市企业进行兼并和重组，培育大型企业集团。完善国有资产监管体系，创新监管模式，提高监管效能和运营效率。二是扎实推进投资、价格体制、交通管理等改革。加强公共资源、资产、资金、资本整合，增强政府调控调节能力。发挥市建投公司融资平台作用，构建政府和企业互补的投融资体系。按照国家和省统一部署，抓好成品油价格和税费改革的落实，做好二级公路收费取消后人员分流工作。加快推进政府投资项目代建制。积极推进资源性产品价格改革。三是深化医药卫生体制等改革。按照国家和省统一部署，积极稳妥推进医药卫生体制改革。加快推进基本医疗保障制度建设，健全基层医疗卫生服务体系，促进基本公共卫生服务均等化。扎实推进事业单位分类改革和金融体制改革，继续推进科技、教育、公用事业、社会管理等领域改革。四是大力发展非公有制经济。认真落实促进非公有制经济发展的各项政策措施，大力抓好融资担保、信用评价、技术服务、人才培训、行业协会等服务体系建设，鼓励支持引导非公有制经济发展。进一步放宽市场准入，积极支持民间资本参与国有企业改革，进入基础设施、公用事业、金融服务和社会事业等领域。引导非公有制企业加快技术进步和管理创新，提升产品档次和企业素质。

大力实施开放带动战略，狠抓招商引资不放松。一是创新招商引资方式。立足我市资源优势和产业布局，按照符合我市产业结构调整、培育优势主导产业、做大做强产业集聚区的需要，主动谋划一批重大项目，实施以产业招商和园区招商为主的定向招商，增强招商引资的针对性和有效性，想方设法争取更多更大的项目落户濮阳。二是优化招商引资平台。优化布局现有产业集聚区，完善配套设施，落实优惠政策，优化投资环境，筑“靓巢”引“金凤”，招大商、引大资，促使园区主导产业上下游企业聚集，形成产业链和产业集群，构筑区域经济发展增长极。三是狠抓项目落实。健全奖励激励机制，完善项目落实机制，重点抓好已签约项目的全程跟踪问效，切实提高合同履约率，做到筛选一个引进一个，落地一个建成一个，使更多的项目在濮阳落地生根，开花结果。

（五）坚持统筹城乡协调发展，不断壮大县域经济实力。一是进一步增强区域性中心城市辐射带动能力。继续实施品牌城市战略，围绕完善城市功能、突出城市特色、增加城市亮点、增强城市现代气息、提升城市品位，进一步加强城市建设和管理。严格城市规划执法，提高城市规划水平。强化城市精细化管理，规范城市秩序，优化城市环境。加大力度，加快城中村改造步伐，逐步将城中村建成基础设施完善、功能齐全、环境优美、物业管理匹配的城市新型社区。抓住国家扩内需保增长和实施适度宽松货币政策的机遇，加强城市道路、供排水、供热等基础设施建设，不断完善公共服务功能，增强综合承载能力。开工建设市城区至工业园区快速通道和市汽车客运总站，加快市第二污水处理厂、城市生活垃圾填埋场等项目建设，完成市西环公园垃圾堆山造景、国电中水回用工程等城市基础设施项目建设。二是加快城乡一体化进程。按照省统一部署和我市第三轮城市总体规划要求，完善各层次的城镇体系规划，逐步形成区域性中心城市、县城、中心镇、居民点协调发展的城乡布局结构。加快体制创新，促进城乡资源共享和生产要素优化配置，逐步构建城乡统一的基础设施、基础产业和公共服务体系。推进市城区至濮阳县城、清丰县城道路等基础设施建设。三是加快发展县域经济。激励、支持县（区）立足本地优势，扩大招商引资，大力发展特色经济，形成主导产业，全面拓展县域经济发展空间。落实扶持政策，完善县域公共基础设施，优化产业布局，促进县域产业集约集群发展，提升县域经济发展的质量和水平。完善政策，下放权力，支持濮阳经济开发区、濮阳工业园区抓招商，上项目，聚产业，创品牌，建成濮阳对外开放的窗口和经济发展的动力基地。

（六）大力发展文化事业和文化产业，加快文化强市建设步伐。一是积极发展文化事业。深入推进文化精品工程和“五个一工程”，推出一批体现时代主旋律的文艺精品。加强公共文化服务体系建设，积极推进新市文化宫建设，重点加强市县两级公共图书馆、博物馆、文化馆建设。实施好农家书屋、自然村广播电视村村通等项目。举办好第六届龙文化节和第三届杂技艺术节。继续做好第三次全国文物普查，保护和利用好我市历史文化遗产。坚持不懈地开展扫黄打非斗争，净化文化市场。二是加快发展文化产业。完善文化产业发展专项规划，落实文化产业扶持政策，在旅游、演艺等领域，集中力量培育龙头文化企业，打造文化品牌。重点整合杂技资源，培育具有国际竞争力的杂技集团。大力发展特色文化产业集群，挖掘春秋文化、濮阳舜帝文化和姓氏文化、清丰孝道、南乐仓颉、范县郑板桥等文化资源，大力推动乒乓球、麦秆画、刀书画、黑陶等特色文化产业发展。完善绿色庄园、濮上园、戚城文物景区、世锦园、单拐等旅游景区基础设施，打造精品旅游线路、旅游演艺品牌和生态休闲旅游园区，提升旅游产业水平。三是切实搞好文化体制改革。加快经营性文化事业单位转企改制。深化公益性文化事业单位内部人事、收入分配和社会保障制度改革。全面完成濮阳日报社印刷厂、市杂技团、市群艺馆3个试点单位改革。拓宽文化产业融资渠道，培育优势民营文化企业。

（七）切实保障和改善民生，推动和谐社会建设。一是千方百计扩大就业。全面落实国家、省促进就业的各项政策，大力推动以创业促就业，支持下岗失业人员再就业，动态消除“零就业家庭”。进一步开发公益性岗位，延长“4050”人员享受公益性岗位就业的各项补贴政策。做好复转军人就业安置工作。二是进一步提高社会保障水平。健全养老保险制度，确保企业离退休人员养老金按时足额发放。完善失业保险制度，确保失业保险金按时足额支付。全面实施被征地农民社会保障制度。扎实推进城镇居民基本医疗保险试点工作，将在校大学生全部纳入城镇居民医保范围。巩固完善新型农村合作医疗制度，提高农民参合率。切实加强社会保障基金监管，保证基金安全。积极推行城乡居民医疗救助制度。完善城乡社会救助体系和优抚保障制度，大力发展社会福利和慈善事业。继续推行和完善住房公积金制度，年内发放贷款1.5亿元。三是大力发展社会事业。优化城乡中小学布局，合理配置公共教育资源，促进城乡义务教育均衡发展。解决好农民工子女在流入地免费接受义务教育问题，认真解决残障儿童特殊教育问题。完善助学制度，使人人享有平等的受教育机会，不让一个孩子因家庭经济困难而失学。实施职业教育攻坚计划，加强市县中等职业学校或职教中心建设，加快发展农村职业教育并逐步实行免费。加快濮阳职业技术学院建设，提高办学水平。增加公共卫生服务经费投入。支持一批县医院、县乡计生服务站、城市社区卫生服务中心建设改造。切实加强中医药、卫生监察和重大疾病控制管理。积极创建白内障无障碍市。深入开展爱国卫生运动，巩固国家卫生城市创建成果。完善计划生育利益导向机制，稳定低生育水平。综合治理出生人口性别比偏高问题，促进人口结构平衡。农村部分计生家庭奖励扶助标准由每人每年600元提高到840元。实施农村妇女孕期防止出生缺陷干预措施，提高出生人口质量。广泛开展全民健身运动，提高竞技体育水平。举办好濮阳市第七届运动会。认真搞好第二次经济普查。全面完成第二次土地调查任务。四是强化安全生产和食品药品安全工作。牢固树立安全发展理念，把人的生命安全放在至高无上的地位。深入开展“安全生产年”活动，全面落实安全生产责任制，重点抓好交通运输、烟花爆竹、危险化学品、建筑施工等重点行业和重点企业的安全监管，抓好商场、学校、娱乐场所等公共聚集地的安全防范，坚决遏制重特大安全事故发生。开展整顿和规范市场秩序专项行动以及“质量和安全年”活动，深入开展食品药品安全专项整治，严肃查处产品质量安全违法违规案件。实行严格的市场准入制度和产品质量追溯、召回制度，让人民群众买得放心、吃得安心、用得舒心。五是维护社会和谐稳定。完善信访评估机制，强化源头治理，着力解决信访问题。积极开展法律援助。深入开展平安创建活动，加强社会治安综合治理，严厉打击各类违法犯罪活动。继续做好民族宗教、外事侨务、残疾人事业、史志、人防、地震、保险、邮政通信等工作。继续抓好对口支援江油市灾后重建工作，按期完成援建任务。

今年，市政府还要积极筹措资金，加大力度，千方百计办好一批关系民生的实事：加强保障性安居工程建设。全市开工建设廉租住房8000平方米，多渠道筹措房源，为符合住房保障条件的城市低收入家庭提供200套廉租住房。扩大廉租住房保障范围，实现保障户数5000户。开工建设经济适用房20万平方米、竣工5万平方米，为城市低收入家庭提供800套经济适用房。加强农村民生工程建设。筹措资金5000万元，解决10万农村居民安全饮水问题。筹措资金5000万元，新建农村户用沼气池2万座。继续实施“万村千乡市场工程”，建设一批农村便民超市和配送中心。提高城乡居民特别是低收入群体收入。继续提高企业退休人员基本养老金水平，人均月增加100元以上。提高城乡低保标准，做到应保尽保。巩固农村五保集中供养率，改善供养条件，年供养标准由1400元提高到1600元。提高优抚对象和建国前老党员抚恤和生活补助标准。认真做好就业再就业工作。实施农村劳动力培训“阳光工程”、扶贫培训“雨露计划”和农村劳动力技能培训计划，分别完成培训1.1万人、1.2万人和2万人。在6个县（区）开展农村劳动力转移就业服务体系基础设施建设试点。鼓励大学生到城乡基层就业，到中小企业和非公有制企业就业或自主创业，建立和完善困难毕业生援助制度。加大扶贫开发力度。完成68个贫困村的整村推进扶贫开发建设任务，确保年内全市再解决4.92万农村贫困人口的脱贫和温饱问题，提高贫困地区和贫困人口的发展能力。大力发展城乡文化事业。免费开放戚城文物景区。开展“舞台艺术送农民”活动，为全市所有乡镇各免费送一场演出。继续实施农村电影放映工程，为全市所有行政村每月公益性放映一场电影。完成15个乡镇文化站扩建改造，完成1290个村级基层服务点改造升级，濮阳县、范县建成国家标准的文化信息共享工程中心。加强全民健身活动场所建设。完成国家、省安排的186个农民体育健身工程。进一步加大教育投入。农村中、小学义务教育学校生均公用经费标准由375元、230元分别提高到500元、300元。继续对农村义务教育阶段学生全部免除学杂费、免费提供教科书，补助农村寄宿制学校家庭经济困难学生生活费。继续改善城乡学校办学条件，实施农村初中校舍改造工程。开工建设第三实验小学教学楼等项目。着力改善群众医疗卫生条件。完成15个中心乡镇卫生院改造，建设51个标准化村卫生室，为52个乡镇卫生院配备基本医疗设备。免费为6000例贫困白内障患者实施复明手术。及时为依法关闭破产企业退休人员办理医保接续。更新购置市人民医院部分医疗设备，年内完成市中医院病房楼投资3500万元，加快市精神病医院扩建等项目建设。扩大免疫规划，在使用5种国家免疫规划疫苗预防7种传染病的基础上，扩展到使用9种疫苗预防12种传染病。加强城市公共基础设施建设。改造10条背街小巷，新建10座垃圾中转站、10座公厕。此外，待省委、省政府今年要办的实事确定后，我市将及时对接，公布实施内容，积极主动抓好落实，确保实事项目圆满完成。

（八）进一步加强精神文明建设，推进民主法制进程。加强社会主义核心价值体系建设，大力弘扬以爱国主义为核心的民族精神和以改革创新为核心的时代精神。加强社会公德、职业道德、家庭美德、个人品德建设，引导人们自觉履行法定义务、社会责任、家庭责任。做好大学生和未成年人思想政治工作。大力开展城乡精神文明创建活动，推进文化、科技、卫生“三下乡”，提高城乡文明程度。深入开展普法教育，提高全民法律素质。坚持依法行政，认真贯彻《行政许可法》、《全面推进依法行政实施纲要》和《关于加强市县政府依法行政的决定》，落实行政执法责任制，强化行政执法监督和责任追究，加强行政复议工作，规范行政执法行为。严格依照法定权限和程序履行职责、接受监督。严格执行人大及其常委会的决议、决定，自觉接受人大及其常委会的法律监督和工作监督；加强与政协的协商，自觉接受政协的民主监督；广泛听取各民主党派、工商联的意见和建议。认真办理人大代表议案、建议和政协提案。充分发挥工会、共青团、妇联等人民团体的桥梁纽带作用。加强社区建设，完善村民自治。加强国防后备力量建设，深入开展国防教育，完善国防动员体系，支持驻濮解放军和武警部队建设，不断提高预备役部队、民兵、人民防空建设质量和国防动员能力。加快民兵预备役训练基地建设。积极开展双拥共建活动，巩固和发展军政军民团结。



三、加强政府自身建设

完成今年各项任务，对新一届政府施政能力是重大考验。我们要主动适应形势发展的新要求，顺应全市人民的新期盼，加强政府自身建设，更好地为发展服务、为基层服务、为群众服务。

（一）解放思想，振奋精神，提高政府应对危机的能力。在当前经济形势异常严峻的特殊时期和加快濮阳发展的关键时期，我们必须进一步解放思想，更新观念，创新思维，以全球视野、战略眼光和大局观念，准确研判国际国内形势变化，深看一层，远看一步，坚持用改革和发展的办法解决前进中的问题与困难，坚定克服困难的信心和决心，保持昂扬向上、奋发有为的精神状态，主动出击，扎实工作，敢于迎难而上，善于捕捉机遇，勇于积极进取，切实提高在复杂形势下做好政府工作的能力，全力保持经济社会平稳较快发展。

（二）转变职能，优化环境，提高政府公共服务能力。以开展学习实践科学发展观活动为契机，按照“两转两提”要求，以提高行政效能和优化发展环境为重点，切实转变职能，强化服务意识，提高服务能力。积极稳妥地推进政府机构改革，上半年完成市级政府机构改革，年底前完成县级政府机构改革。积极推进行政管理体制改革，强化社会管理和公共服务职能。深化行政审批制度改革，减少和规范行政审批事项，进一步减少行政事业性收费。深入推行政务公开，进一步加强行政服务中心建设，促进政府工作公正、公平、便民。大力推行效能问责制，深入实施“治庸计划”，加大责任追究力度，着力解决行政不作为、乱作为、不会作为问题，严肃查处影响企业发展的违纪违法事件，树立濮阳改革开放的新形象。

（三）求真务实，转变作风，提高政府执行力。坚持深入实际、深入基层、深入群众，认真开展调查研究，虚心问政于民、问需于民、问计于民，努力使发展目标、工作思路、政策措施更加符合实际、更加符合人民群众的愿望；贯彻民主集中制原则，完善重大事项决策规则和程序，不断提高科学决策水平；健全抓落实的工作机制，严格实行目标责任制和责任追究制，明确责任，强化督导，落实奖惩，全力以赴抓好各项决策部署的落实，确保定一件干一件，干一件成一件，切实提高政府执行力。

（四）勤政为民，廉洁从政，提高政府公信力。牢固树立宗旨意识，把濮阳发展和百姓福祉作为政府工作研究、决策的出发点和落脚点，常怀为民之心，勤思富民之策，践行惠民之举，多解决一些影响改革发展稳定的难事，多干一些打基础利长远的实事，多办一些解民忧惠民生的好事。厉行节约，勤俭办事，反对奢侈浪费，把有限的资金资源、财力物力集中用到促进科学发展上，用到为人民谋利益上。严格控制新建楼堂馆所。今年公务购车和用车、会议、公务接待、党政机关出国（境）等经费要按规定从严控制，予以削减。强化审计监督，严格行政监察，形成用制度管权、管事、管人的有效机制。严格落实党风廉政建设责任制，健全惩治和预防腐败体系。加强公务员队伍建设，以务实清廉的良好形象取信于民，团结和带领全市人民战胜困难，努力创造经济社会发展新业绩。

各位代表，困难和挑战考验着我们，责任和使命激励着我们。让我们在省委、省政府和市委的坚强领导下，以邓小平理论和“三个代表”重要思想为指导，深入贯彻落实科学发展观，团结奋进，扎实工作，以优异成绩向新中国成立60周年献礼！我们坚信，经过五年的努力，濮阳的经济将更具活力，文化将更加繁荣，社会将更加和谐，环境将更加优美，人民将更加富足，我们富裕文明和谐濮阳的奋斗目标一定能够实现，我们濮阳的明天一定会更加美好！
